# Особенности вариабельности гликемии у мужчин с разными типами ожирения

**DOI:** 10.14341/probl13416

**Published:** 2024-03-06

**Authors:** М. Ю. Сорокин, Б. Б. Пинхасов, Ю. В. Лутов, В. Г. Селятицкая

**Affiliations:** Федеральный исследовательский центр фундаментальной и трансляционной медицины; Федеральный исследовательский центр фундаментальной и трансляционной медицины; Новосибирский государственный медицинский университет; Федеральный исследовательский центр фундаментальной и трансляционной медицины; Федеральный исследовательский центр фундаментальной и трансляционной медицины

**Keywords:** вариабельность гликемии, непрерывный мониторинг уровня глюкозы, ожирение, подкожный тип распределения жира, абдоминальный тип распределения жира, инсулинорезистентность

## Abstract

**ОБОСНОВАНИЕ:**

ОБОСНОВАНИЕ. Ожирение во многом предопределяет метаболическую основу развития сахарного диабета 2 типа (СД2), в связи с чем актуальным является анализ вариабельности гликемии у лиц с ожирением, особенно при его разных фенотипах.

**ЦЕЛЬ:**

ЦЕЛЬ. Изучить особенности вариабельности гликемии у мужчин с разной топографией распределения жировой ткани в условиях привычного рациона.

**МАТЕРИАЛЫ И МЕТОДЫ:**

МАТЕРИАЛЫ И МЕТОДЫ. В исследовании участвовали мужчины в возрасте от 25 до 65 лет. Общее число участников составило 43 мужчины. Группу 1 (n=17) составили мужчины с ожирением и подкожным типом распределения жира (ПТРЖ), группу 2 (n=16) — мужчины с ожирением и абдоминальным типом распределения жира (АТРЖ). В группу 3 (сравнения) вошли 10 мужчин с нормальной массой тела (НМТ). Проведен 2-дневный непрерывный мониторинг уровня глюкозы (НМГ), в эти дни режим питания, физических нагрузок и трудовой деятельности не отличался от привычного. Анализировали параметры, индексы и отношения, характеризующие вариабельность гликемии (ВГ), которые рассчитывали для дневных (06:00–23:59) и ночных (00:00–05:59) часов.

**РЕЗУЛЬТАТЫ:**

РЕЗУЛЬТАТЫ. Анализ ряда ключевых показателей и индексов, характеризующих ВГ в течение дня и ночи, у мужчин с НМТ и ожирением без учета типа распределения жира не выявил статистически значимых различий. Разделение мужчин с ожирением на группы с АТРЖ и ПТРЖ показало, что мужчины с АТРЖ имели статистически значимо более высокий средний уровень глюкозы, стандартное отклонение уровня гликемии и коэффициент вариации; также статистически значимые различия были получены относительно индекса CONGA и J-индекса. Анализ индексов LBGI и HBGI, отражающих риски развития соответственно гипогликемии и гипергликемии, показал, что индекс LBGI был более высокий у мужчин с ПТРЖ, в то время как индекс НBGI — у мужчин с АТРЖ. Сравнительный анализ величин дневных и ночных показателей, характеризующих ВГ, показал, что величины дневных показателей были статистически значимо выше относительно ночных показателей у мужчин как с НМТ, так и с ожирением. При этом средние уровни глюкозы у мужчин с НМТ в дневное и ночное время не различались между собой, у мужчин с АТРЖ в ночное время снижение уровня глюкозы отмечено на уровне тенденции (р=0,08), а у мужчин с ПТРЖ оно оказалось статистически значимым (р=0,005).

**ЗАКЛЮЧЕНИЕ:**

ЗАКЛЮЧЕНИЕ. Оценка ВГ у мужчин с ожирением позволяет говорить, что наличие абдоминального или подкожного типов распределения жировой ткани ассоциировано с конкретными особенностями регуляции углеводного обмена и определяет разный уровень риска развития СД2 при ПТРЖ и АТРЖ.

## Abstract (en)

**BACKGROUND:**

BACKGROUND: Obesity generally determines the metabolic basis for the development of type 2 diabetes. Therefore the analysis of glycemic variability in obese individuals, especially in its different phenotypes, acquires particular relevance.

**AIM:**

AIM: To investigate the features of glycemic variability in men with different adipose tissue distribution topography within usual dietary conditions.

**MATERIALS AND METHODS:**

MATERIALS AND METHODS: The study enrolled 43 men aged 25-65 years. Group 1 (n=17) represented obese men with subcutaneous fat distribution (SFD) while group 2 (n=16) consisted of obese men with abdominal fat distribution (AFD) and group 3 (comparator) included 10 male subjects with normal body weight (NBW). A 2-day continuous glucose monitoring (CGM) under condition of usual diet, work and physical activity was performed in each study subject. A number of parameters, indices and ratios had been assessed describing glycemic variability (GV) for daytime (6.00–23.59) and night (0.00–5.59) hours.

**RESULTS:**

RESULTS: Comparative analysis of key parameters and indices describing daytime and night GV in NBW and obese men without fat distribution adjustment did not reveal statistically significant differences. After fat distribution adjustment  significantly higher mean glucose levels, standard deviation of glycemic levels and coefficient of variation were found in AFD group; also statistically significant differences were revealed in CONGA index and J-index. An analysis of the LBGI and HBGI indices that are respectively reflecting the risks of hypo- and hyperglycemia showed that the LBGI index was higher in obese men with SFD while the НBGI index was higher in men with AFD. A comparative analysis of GV parameters showed that daytime indicators values were significantly higher relative to nighttime. However the ambiguous changes in the mean glucose levels was found between study groups. Specifically in NBW men daytime and nighttime glycemia didn’t differ, whereas in AFD group there was a trend to decrease in night glucose levels (p = 0.08) while in men with SFD night decrease in glycemia became statistically significant (p=0.005).

**CONCLUSION:**

CONCLUSION: Results of glycemic variability assessment in obese men suggest that abdominal and subcutaneous types of fat distribution are associated with specific features of carbohydrate metabolism and determine different risk levels for developing type 2 diabetes in patients with AFD and SFD.

## ОБОСНОВАНИЕ

В последние годы в качестве инструмента для исследования гомеостаза глюкозы широкое применение нашла технология непрерывного мониторинга уровня глюкозы (НМГ), когда исследователь, врач и пациент получают информацию об уровне глюкозы с 5-минутным интервалом на протяжении нескольких суток [1]. Наиболее часто данная технология применяется для оценки вариабельности глюкозы у пациентов с сахарным диабетом (СД) [2]. НМГ позволяет контролировать вариабельность гликемии, а следовательно, повышает эффективность проводимой терапии, что, в свою очередь, снижает риски развития как гипогликемических состояний, так и гипергликемии, а значит, и связанных с ней микро- и макрососудистых осложнений [1][2]. Гипергликемия при диабете характеризуется не только стойким повышением уровня сахара, но и выраженной вариабельностью гликемии (ВГ), оценка которой часто используется для выявления потенциальных клинических проблем у пациентов с диабетом [3].

Установлена связь между выраженностью колебаний гликемии и формированием осложнений и исходов СД. Убедительные данные свидетельствуют о том, что резкие колебания уровня глюкозы вокруг среднего значения в течение ежедневного периода интермиттирующей гипергликемии могут играть важную роль в развитии сердечно-сосудистых заболеваний у пациентов с СД 2 типа (СД2). Известно, что ВГ порождает окислительный стресс и потенциально способствует развитию как макро-, так и микрососудистых осложнений при диабете [4]. Эффекты значительного подъема уровня глюкозы опосредованы через усиление продукции свободных радикалов и сохраняются на протяжении нескольких дней последующей нормогликемии [5]. Представляется вероятным, что «пики» гипергликемии могут индуцировать длительные, самоподдерживающиеся процессы в сосудах: окислительный стресс и хроническое воспаление, играющие важную роль в феномене «метаболической памяти» и развитии диабетических ангиопатий [6][7]. Следовательно, выраженность колебаний уровня глюкозы можно использовать не только как показатель эффективности терапии СД, но и как показатель риска развития СД и его осложнений.

Если рассматривать ожирение как метаболическую основу развития СД2, то возникает вопрос: как будет вести себя ВГ при ожирении? Как говорилось выше, большинство работ по исследованию ВГ на основании НМГ посвящено пациентам с СД, однако имеются исследования по ВГ у лиц с нормальной массой тела [1] и ожирением [8]. Было определено, что при ожирении параметры ВГ не отличались от таковых у лиц с нормальной массой тела [9]. Такие результаты могут быть связаны не столько с истинным отсутствием различий при нормальной массе тела и ожирении, сколько с гетерогенностью ожирения, а именно с наличием в популяции лиц с метаболически здоровым (МЗО) и нездоровым (МНЗО) ожирением [10]. Эти два разных по топографии накопления жировой ткани типа ожирения проявляют по многим параметрам противоположные результаты. Например, жировые депо различных анатомических областей существенно различаются по метаболической, гормональной, адипокиновой, цитокиновой активности [11]. Исследований по изучению ВГ у лиц с разными типами ожирения мы в современной научно-медицинской литературе не нашли [12].

## ЦЕЛЬ ИССЛЕДОВАНИЯ

Изучить особенности вариабельности гликемии у мужчин с разной топографией распределения жировой ткани в условиях привычного рациона.

## МАТЕРИАЛЫ И МЕТОДЫ

## Место и время проведения исследования

Место проведения. Клиника Федерального исследовательского центра фундаментальной и трансляционной медицины (Новосибирск).

Время исследования. Набор материала продолжался с января 2022-го по май 2023 гг.

## Изучаемые популяции.

Популяция. В исследовании участвовали 2 группы мужчин в возрасте от 25 до 65 лет, группа 1 — мужчины с ожирением, группа 2 — мужчины без ожирения.

Критерии включения: 1) первичное ожирение алиментарно-конституциональной природы, 2) мужской пол, 3) возраст от 25 до 65 лет, 4) готовность воздерживаться от употребления алкоголя в период участия в исследовании, 5) соблюдать привычный рацион и режим питания, 6) для лиц без ожирения индекс массы тела (ИМТ) <25 кг/м², для лиц с ожирением ИМТ≥30 кг/м².

Критерии исключения: 1) вторичное ожирение, 2) избыточная масса тела ИМТ≥25 кг/м² и <30 кг/м², 3) наличие диагностированного ранее СД, 4) наличие сопутствующих эндокринологических заболеваний, 5) прием гормональных препаратов, сахароснижающих препаратов, 6) сменная работа.

## Способ формирования выборки из изучаемой популяции (или нескольких выборок из нескольких изучаемых популяций)

Выборка здоровых мужчин формировалась произвольным образом из числа сотрудников ФИЦ ФМТ. Выборка мужчин с ожирением представлена пациентами, которые приходили на консультативный прием к врачу эндокринологу с проблемой ожирения.

Дизайн исследования: одноцентровое интервенционное одномоментное (поперечное) исследование на одной популяции.

## Описание медицинского вмешательства (для интервенционных исследований)

У всех участников исследования после подписания информированного согласия проводили сбор анамнеза, клинический осмотр эндокринолога, измерение антропометрических показателей. При соответствии критериям включения и отсутствии критериев исключения проводили 2-дневную процедуру НМГ [13].

## Методы

Исследуемым мужчинам проводили антропометрическое обследование с измерением массы тела (кг), роста (м), окружности талии (см) и бедер (см). Рассчитывали индекс массы тела (ИМТ) как отношение массы тела (кг) к росту (м²). По критериям ВОЗ ожирению соответствует ИМТ≥30 кг/м², нормальной массе тела (НМТ) — менее 25 кг/м². Тип ожирения оценивали по соотношению окружности талии к окружности бедер (ОТ/ОБ). При ОТ/ОБ<0,95 мужчин относили к группе с подкожным типом распределения жира (ПТРЖ), при ОТ/ОБ≥0,95 — к группе с абдоминальным типом распределения жира (АТРЖ) [14]. В группу (сравнения) вошли 10 мужчин с НМТ.

НМГ проводили с помощью системы для мониторирования iPro2 и программного обеспечения CareLink® iPro (Medtronic, США). Длительность НМГ составляла 2 дня, в которые режим питания, физических нагрузок и трудовой деятельности не отличался от привычного. Исследуемым предоставляли инструкции по правилам калибровки и другим аспектам процедуры мониторирования. Калибровка осуществлялась глюкометрами OneTouch® Verio®Pro+ и тест-полосками к этим глюкометрам. На основании данных НМГ проводили расчет следующих параметров: средний уровень глюкозы, стандартное отклонение (Standard Deviation, SD), коэффициент вариабельности (Coefficient of Variation, CV), средняя амплитуда колебаний гликемии (Mean Amplitude of Glycemic Excursions, MAGE), 2-часовой индекс длительного повышения гликемии (Сontinuous Overlapping Net Glycemic Action, CONGA), индекс лабильности (Lability Index, LI), J-индекс, скорость изменений уровня глюкозы (Mean Absolute Glucose rate of change, MAG), М-value, индекс риска гипергликемии (High Blood Glucose Index, HBGI), индекс риска гипогликемии (Low Blood Glucose Index, LBGI). В данной панели индексов SD и СV отражают разброс значений уровня глюкозы, MAGE оценивает амплитуду колебаний, MAG — скорость изменения уровня глюкозы, LBGI и HBGI — риск чрезмерно высокого или низкого уровня глюкозы соответственно, CONGA, J-индекс, LI и M-value являются мерой качества контроля гликемии и ВГ [14]. Расчет параметров ВГ проводили с использованием калькулятора EasyGV v. 9.0, предложенного N. Hill и соавт. [1][15]. Все показатели рассчитывали для суточных записей, дневных (06:00–23:59) и ночных (00:00–05:59) часов.

## Статистический анализ

Статистический анализ проведен с использованием программы STATISTICA 10,0 (StatSoftInc, 2011, CША). Нормальность распределения проверяли с помощью критерия Колмогорова-Смирнова. Поскольку распределение большинства изученных признаков было отличным от нормального, применяли методы непараметрической статистики. Проверку гипотезы о равенстве генеральных средних в сравниваемых группах проводили с помощью непараметрического критерия Манна–Уитни, внутригрупповые различия оценивали с использованием критерия Вилкоксона. Минимальную вероятность справедливости нулевой гипотезы принимали при 5% уровне значимости (р<0,05). При описании количественных признаков использовали величины медианы, 25-го и 75-го процентилей.

## Этическая экспертиза

Проведение исследования было одобрено локальным Комитетом по биомедицинской этике Федерального исследовательского центра фундаментальной и трансляционной медицины (заключение № 23/1 от 09.12.2021). С пациентами проводили беседу, объясняющую цель и задачи исследования; от них было получено информированное согласие на участие в исследовании.

## РЕЗУЛЬТАТЫ

Общее число участников исследования составило 43 мужчины, из них 10 мужчин с НМТ и 33 мужчины с ожирением. Сравнительный анализ возраста и основных антропометрических показателей, характеризующих выраженность ожирения, приведен в таблице 1. Показано, что у мужчин с ожирением все показатели, отражающие количество жировой ткани, существенно выше относительно мужчин, имеющих НМТ, однако показатель, характеризующий топографию жировой ткани (отношение ОТ/ОБ), у мужчин с НМТ и ожирением значимо не различался.

**Table:** Таблица 1. Сравнительная характеристика возраста и основных антропометрических показателей у мужчин в группах с нормальной массой тела и ожирением Ме (LQ; UQ)

Показатель	Группа мужчин с нормальной массой тела, n=10	Группа мужчин с ожирением, n=33	р
Возраст, лет	35,0 [ 35,0–50,0]	38,0 [ 30,0–51,0]	0,676
Вес, кг	74,4 [ 73,0–76,8]	106,0 [ 90,0–117,3]	0,000
ИМТ, кг/м²	24,3 [ 23,2–24,8]	32,1 [ 30,5–36,5]	0,000
ОТ, см	79,0 [ 78,0–87,5]	105,0 [ 99,5–120,0]	0,000
ОБ, см	83,0 [ 81,5–98,0]	114,0 [ 106,5–117,0]	0,000
ОТ/ОБ, у.е.	0,95 [ 0,91–0,95]	0,94 [ 0,92–1,00]	0,211
Жир, %	17,2 [ 16,8–18,1]	27,1 [ 23,5–34,0]	0,000
Жир, кг	12,6 [ 12,2–13,9]	26,2 [ 22,5–36,2]	0,000

Анализ ряда ключевых показателей и индексов, характеризующих ВГ в течение дня и ночи, у мужчин с НМТ и ожирением не выявил статистически значимых различий между ними (табл. 2). Сравнительный анализ величин дневных и ночных показателей, характеризующих ВГ в группах, показал, что величины дневных показателей были статистически значимо выше относительно ночных показателей у мужчин как с НМТ, так и с ожирением, за исключением среднего уровня глюкозы и индекса CONGA у мужчин с НМТ (табл. 2).

**Table:** Таблица 2. Дневные и ночные значения параметров вариабельности глюкозы у мужчин в группах с нормальной массой тела и ожирением Ме (LQ; UQ) Примечание. * — р<0,05; ** — p<0,005; *** — p<0,001, уровень статистически значимых различий между дневными и ночными показателями.

Показатель	Группа мужчин с нормальной массой тела, n=10	Группа мужчин с ожирением, n=33	р
Дневные показатели ВГ
Cредний уровень глюкозы, ммоль/л	5,47 [ 5,47–5,98]	5,89 [ 5,33–6,55]	0,514
SD, ммоль/л	0,46 [ 0,46–0,83]	0,64 [ 0,45–1,08]	0,534
CV, %	9,05 [ 8,26–11,67]	10,60 [ 7,93–13,96]	0,587
MAGE, ммоль/л	1,35 [ 1,05–1,46]	1,50 [ 0,90–2,85]	0,537
CONGA, ммоль/л	5,10 [ 5,10–5,32]	5,40 [ 4,95–5,96]	0,574
LI, (ммоль/л) 2/ч	0,33 [ 0,33–1,28]	0,55 [ 0,26–1,57]	0,773
J-индекс, (ммоль/л)²	11,41 [ 11,41–15,22]	14,12 [ 11,11–19,10]	0,595
LBGI, у.е.	1,02 [ 0,83–1,02]	1,14 [ 0,26–1,50]	0,595
HBGI, у.е.	0,07 [ 0,07–1,12]	0,41 [ 0,09–1,36]	0,457
M-VALUE, у.е.	1,14 [ 1,04–1,14]	1,43 [ 0,54–1,74]	0,704
MAG, ммоль/л/ч	0,80 [ 0,80–1,27]	0,93 [ 0,64–1,27]	0,988
Ночные показатели ВГ
Cредний уровень глюкозы, ммоль/л	5,61 [ 5,61–5,70]	5,58 [ 4,98–6,19]***	0,891
SD, ммоль/л	0,32 [ 0,32–0,32]**	0,32 [ 0,19–0,51]***	0,773
CV, %	6,16 [ 4,12–6,99]**	5,4 [ 3,12–7,72]***	0,312
MAGE	0,73 [ 0,67–0,94]**	5,20 [ 4,84–5,74]***	0,682
CONGA, ммоль/л	5,26 [ 5,26–5,26]	0,65 [ 0,45–1,10]*	0,188
LI, (ммоль/л) 2/ч	0,22 [ 0,22–0,22]**	0,18 [ 0,08–0,46]**	0,404
J-индекс, (ммоль/л)²	11,40 [ 9,41–11,67]**	11,64 [ 8,78–14,49]***	0,940
LBGI, у.е.	0,61 [ 0,61–0,82]*	0,94 [ 0,26–2,22]*	0,268
HBGI, у.е.	0,02 [ 0,02–0,09]**	0,02 [ 0,00–0,60]***	0,704
M-VALUE, у.е.	0,68 [ 0,68–0,76]*	1,23 [ 0,49–2,83]*	0,062
MAG, ммоль/л/ч	0,67 [ 0,67–0,67]**	0,56 [ 0,36–0,96]***	0,773

Для выявления особенностей ВГ, ассоциированных с типом распределения жира у мужчин с ожирением, их разделили на группы с подкожным и абдоминальным типами распределения жира. Группу 1 (n=17) составили мужчины с подкожным типом распределения жира (ПТРЖ), группу 2 (n=16) — мужчины с абдоминальным типом распределения жира (АТРЖ).

Разделение мужчин с ожирением на подгруппы с АТРЖ и ПТРЖ позволило выявить существенные различия как по ряду антропометрических показателей (табл. 3), так и по особенностям показателей ВГ (табл. 4).

**Table:** Таблица 3. Сравнительная характеристика возраста и основных антропометрических показателей у мужчин в группах с нормальной массой тела и разными типами распределения жира при ожирении Ме (LQ; UQ) Примечание. АТРЖ — абдоминальный тип распределения жира, ПТРЖ — подкожный тип распределения жира.

Показатель	Группа мужчин с нормальной массой тела, n=10	Группа мужчин с ожирением и АТРЖ, n=16	Группа мужчин с ожирением и ПТРЖ, n=17	р
1	2	3	1–2	1–3	2–3
Возраст, лет	35,0 [ 35,0–50,0]	40,4 [ 37,5–53,0]	36,0 [ 30,0–51,0]	0,135	0,509	0,293
Вес, кг	74,4 [ 73,0–76,8]	112,0 [ 88,0–123,0]	106,5 [ 91,0–112,0]	0,000	0,000	0,387
ИМТ, кг/м²	24,3 [ 23,2–24,8]	35,4 [ 30,7–37,9]	31,2 [ 30,2–32,5]	0,000	0,000	0,167
ОТ, см	79,0 [ 78,0–87,5]	118,5 [ 100,5–122,0]	99,0 [ 97,0–105,5]	0,000	0,000	0,006
ОБ, см	83,0 [ 81,5–98,0]	115,0 [ 102,0–118,5]	114,0 [ 106,0– 116,0]	0,000	0,000	0,801
ОТ/ОБ, у.е.	0,95 [ 0,91–0,95]	1,01 [ 0,98–1,04]	0,92 [ 0,89–0,93]	0,000	0,071	0,000
Жир, %	17,2 [ 16,8–18,1]	33,5 [ 26,0–39,6]	27,1 [ 22,0–30,9]	0,000	0,000	0,061
Жир, кг	12,6 [ 12,2–13,9]	35,2 [ 26,1–51,5]	26,7 [ 21,8–31,7]	0,000	0,000	0,059

**Table:** Таблица 4. Дневные и ночные значения параметров вариабельности глюкозы у мужчин в группах с нормальной массой тела и разными типами распределения жира при ожирении Ме (LQ; UQ) Примечание. АТРЖ — абдоминальный тип распределения жира, ПТРЖ — подкожный тип распределения жира; # — p<0,08; * — р <0,05; ** — p<0,005, уровень статистически значимых различий между дневными и ночными показателями.

Показатель	Группа мужчин с нормальной массой тела, n=10	Группа мужчин с ожирением и АТРЖ, n=16	Группа мужчин с ожирением и ПТРЖ, n=17	р
1	2	3	1–2	1–3	2–3
Дневные показатели ВГ
Cредний уровень глюкозы, ммоль/л	5,47 [ 5,47–5,98]	6,50 [ 5,65–6,74]	5,62 [ 5,27–5,96]	0,096	0,598	0,034
SD, ммоль/л	0,46 [ 0,46–0,83]	0,88 [ 0,54–1,12]	0,50 [ 0,41–0,93]	0,119	0,637	0,050
CV, %	9,05 [ 8,26–11,67]	13,11 [ 9,27–16,54]	9,29 [ 6,72–11,37]	0,015	0,452	0,001
MAGE, ммоль/л	1,35 [ 1,05–1,46]	1,77 [ 0,90–3,25]	1,30 [ 0,93–2,04]	0,162	0,802	0,313
CONGA, ммоль/л	5,10 [ 5,10–5,32]	5,67 [ 5,20–6,10]	5,09 [ 4,85–5,65]	0,077	0,421	0,050
LI, (ммоль/л) 2/ч	0,33 [ 0,33–1,28]	0,69 [ 0,31–1,94]	0,39 [ 0,22–0,99]	0,444	0,802	0,161
J-индекс, (ммоль/л)²	11,41 [ 11,41–15,22]	18,15 [ 11,78–19,47]	12,66 [ 10,85–15,95]	0,215	0,760	0,031
LBGI, у.е.	1,02 [ 0,83–1,02]	0,37 [ 0,17–1,51]	1,35 [ 0,66–1,50]	0,654	0,141	0,173
HBGI, у.е.	0,07 [ 0,07–1,12]	1,06 [ 0,09–1,46]	0,35 [ 0,09–0,97]	0,384	0,677	0,213
M-VALUE, у.е.	1,14 [ 1,04–1,14]	0,89 [ 0,40–1,89]	1,53 [ 0,93–1,74]	0,895	0,389	0,621
MAG, ммоль/л/ч	0,80 [ 0,80–1,27]	1,06 [ 0,71–1,69]	0,85 [ 0,56–1,05]	0,853	0,802	0,149
Ночные показатели ВГ
Cредний уровень глюкозы, ммоль/л	5,61 [ 5,61–5,70]	5,99 [ 5,68–6,42]#	5,17 [ 4,97–5,58]**	0,037	0,014	0,011
SD, ммоль/л	0,32 [ 0,32–0,32]**	0,38 [ 0,24–0,62]**	0,28 [ 0,17–0,34]**	0,414	0,157	0,086
CV, %	6,16 [ 4,12–6,99]**	5,87 [ 3,69–8,36]**	4,34 [ 3,05–6,13]**	0,854	0,034	0,096
MAGE, ммоль/л	0,73 [ 0,67–0,94]**	0,81 [ 0,54–1,20]**	0,49 [ 0,40–0,76]**	0,853	0,048	0,038
CONGA, ммоль/л	5,26 [ 5,26–5,26]	5,58 [ 5,23–6,10]	4,88 [ 4,80–5,20]*	0,108	0,014	0,008
LI, (ммоль/л) 2/ч	0,22 [ 0,22–0,22]**	0,24 [ 0,14–0,57]*	0,10 [ 0,07–0,25]#	0,979	0,114	0,093
J-индекс, (ммоль/л)²	11,41 [ 9,41–11,67]**	13,31 [ 11,22–15,79]**	9,83 [ 8,70–11,74]**	0,163	0,102	0,034
LBGI, у.е.	0,61 [ 0,61–0,82]*	0,40 [ 0,17–0,83]	1,64 [ 0,96–2,66]**	0,197	0,001	0,007
HBGI, у.е.	0,02 [ 0,02–0,09]**	0,20 [ 0,01–0,85]*	0,01 [ 0,00–0,02]**	0,236	0,049	0,048
M-VALUE, у.е.	0,68 [ 0,68–0,76]*	0,71 [ 0,29–1,97]	1,92 [ 1,10–3,22]**	0,937	0,000	0,050
MAG, ммоль/л/ч	0,67 [ 0,67–0,67]**	0,69 [ 0,42–1,17]**	0,40 [ 0,36–0,78]**	0,617	0,279	0,220

Показано, что, хотя мужчины с АТРЖ и ПТРЖ статистически значимо не различались по массе тела и ИМТ, мужчины с АТРЖ имели более высокие показатели относительного и абсолютного количества жировой ткани в организме. Наиболее выражено это увеличение проявилось накоплением жировой ткани в абдоминальной области, о чем свидетельствовали величины показателя ОТ, а также отношения ОТ/ОБ (табл. 3).

Анализ показателей и индексов, характеризующих ВГ у лиц с разными типами распределения жира в течение дня и ночи, представлен в таблице 4. Показано, что в течение дня мужчины с АТРЖ имели статистически значимо более высокий средний уровень глюкозы, стандартное отклонение уровня гликемии и коэффициент вариации, отражающий процент от среднего значения гликемии. Также статистически значимые различия были найдены относительно индекса CONGA, характеризующего величину дисперсии (SD). Были выявлены различия J-индекса, который был выше у мужчин с АТРЖ. Считается, что данный индекс свидетельствует о гликемическом контроле и в норме находится в диапазоне 10–30, превышение же данного индекса более 30 свидетельствует о плохом контроле, а свыше 40 — о его потере. Относительно индексов LBGI и HBGI, отражающих риски развития гипогликемии и гипергликемии соответственно, показано, что величина индекса LBGI была более высокой у мужчин с ПТРЖ, в то время как величина индекса НBGI — у мужчин с АТРЖ. По другим индексам LI и MAG, которые преимущественно используются для характеристики гликемии у лиц, получающих инсулин, статистически значимых различий не было выявлено, и они находились в пределах референсных значений.

Ночные показатели, характеризующие ВГ, также выявили межгрупповые статистически значимые различия, а именно средний уровень глюкозы, индекс CONGA, J-индекс, M-Value, показатели SD, CV, LI различались на уровне тенденции, при этом различия индексов LBGI и HBGI, характеризующих риски развития гипо- и гипергликемии, достигли статистической значимости, показав, что мужчины с ПТРЖ имеют склонность к развитию гипогликемических состояний, а мужчины с АТРЖ — гипергликемических. Более того, в ночное время была отмечена статистически значимая разница анализируемых показателей и индексов между группами мужчин с ПТРЖ и мужчин, имеющих НМТ. Мужчины с ПТРЖ имели статистически значимо более низкие величины следующих показателей: среднего уровня глюкозы, СV, CONGA, M-Value, а также индекса HBGI, в то время как индекс LBGI у них был существенно выше.

Сравнительный анализ величин дневных и ночных показателей, характеризующих ВГ, показал, что величины дневных показателей были статистически значимо выше относительно ночных показателей у мужчин как с НМТ, так и с ожирением. При этом средние уровни глюкозы у мужчин с НМТ в дневное и ночное время не различались между собой, у мужчин с АТРЖ в ночное время снижение уровня глюкозы отмечено на уровне тенденции (р=0,08), а у мужчин с ПТРЖ оно оказалось статистически значимым (р=0,005).

## ОБСУЖДЕНИЕ

## Сопоставление с другими публикациями

Проблема гетерогенности ожирения начинается с разработки критериев отнесения ожирения к МЗО или МНЗО [16]. Использование для мужчин граничного значения, предложенного ВОЗ для выявления метаболического синдрома, а Российским кардиологическим обществом — для выявления МНЗО, а именно ОТ/ОБ>0,9 у.е., является крайне жестким условием, и более 95% мужчин с ожирением будут отнесены к МНЗО, что практически сводит на нет использование данного показателя, изначально направленного на выделение метаболических фенотипов, ассоциированных с гетерогенностью жировой ткани. Ранее мы показали, что для решения этой задачи наиболее информативным граничным для мужчин является показатель ОТ/ОБ — 0,95 у.е., а для женщин ОТ/ОБ — 0,85 у.е. [14]. При адекватных граничных критериях отношения ОТ/ОБ гетерогенность жировой ткани начинает явно проявляться.

Относительно изучения особенностей суточных ритмов углеводного обмена с использованием теста толерантности к глюкозе у женщин с разными типами ожирения было показано, что у женщин с гиноидным ожирением (подкожный тип распределения жира) прием глюкозы сопровождался относительной гиперинсулинемией и последующей постпрандиальной гипогликемией, а у женщин с андроидным ожирением (абдоминальный тип распределения жира) была выявлена абсолютная гиперинсулинемия с инсулинорезистентностью вне зависимости от времени суток [17].

В другом исследовании регуляции углеводно-жирового обмена показано, что у женщин с андроидным ожирением уже на начальных этапах накопления висцеральных жировых депо начинается перестройка метаболизма на липидный тип обмена, т.е. СЖК становятся предпочитаемым субстратом окисления, тем самым вытесняя глюкозу из энергообмена. Ответная реакция со стороны гормональной регуляции при такой перестройке характеризуется повышением уровня инсулина. Усиление активности ГГНС и, как следствие, увеличение при этом уровня кортизола, еще более способствуют развитию инсулинорезистентности. Реакция на пищевую депривацию при таком типе ожирения проявляется сочетанным снижением уровней как инсулина, так и кортизола в крови, что свидетельствует о тесной связи этих гормонов в формировании абдоминального типа распределения жира. В то же время у женщин с гиноидным ожирением реакция на пищевую депривацию проявляется увеличением уровня кортизола в крови и стрессорной активацией процессов глюконеогенеза, направленного на поддержание необходимого уровня глюкозы — ведущего субстрата окисления при данном типе ожирения [18].

Результаты настоящего исследования показали, что при сравнении показателей и индексов, характеризующих ВГ у мужчин с НМТ и ожирением без подразделения их на группы с ПТРЖ и АТРЖ, отсутствовали статистически значимые различия. Данные результаты совпадают с работой [9], в которой авторы тоже не нашли различий ВГ между мужчинами и женщинами с НМТ и лицами, имеющими абдоминальное ожирение. При этом инсулин натощак и HOMA-IR были значительно выше у лиц с ожирением. Более того, исследователи указывают: их удивило, что стандартное отклонение уровня глюкозы в крови, хотя и не было статистически значимым, было выше у участников без ожирения. В другой работе средний уровень глюкозы, SD и MAGE были статистически значимо более высокими у мужчин с абдоминальным ожирением относительно мужчин с НМТ [3].

После разделения мужчин на группы с абдоминальным и подкожным типами распределения жира были выявлены значимые различия части исследуемых показателей не только между этими группами, но и по сравнению с мужчинами, имеющими НМТ. Обращает внимание, что мужчины с АТРЖ относительно мужчин с ПТРЖ имели более высокий уровень глюкозы и показатели характеризующие ВГ (SD, CV%, LI, CONGA другие индексы) как в дневные, так и особенно в ночные часы, а относительно мужчин с НМТ отмечено лишь повышение CV% в дневные часы и среднего уровня глюкозы ночью. Интересной является разнонаправленность индексов LBGI и НBGI, отражающих риски развития гипо- и гипергликемических состояний. Если риск развития гипогликемии в большей степени имеют мужчины с ПТРЖ, то риск гипергликемии — мужчины с АТРЖ.

## Клиническая значимость результатов

Показано, что для выявления особенностей патогенеза нарушений углеводного обмена пациентов с ожирением необходимо разделять на группы с разными фенотипами ожирения с использованием отношения ОТ/ОБ и его граничным значением для мужчин — 0,95 у.е., в ином случае может наблюдается нивелирование различий показателей при сравнении с лицами без ожирения.

## Ограничения исследования

Ограничением исследования является малый объем выборки, включение в исследование только лиц мужского пола. В исследовании не принимали во внимание возможность наличия в группах обследованных пациентов лиц с саркопеническим ожирением, однако, судя по данным научно-медицинской литературы [19], вероятность этого достаточно низка, поскольку саркопеническое ожирение развивается преимущественно в пожилом и старческом возрастах, а в проведенном исследовании количество мужчин старше 55 лет составило всего 3 человека, а медиана возраста в группах мужчин с ожирением 38,0 [ 30,0–51,0] лет.

## Направления дальнейших исследований

С учетом полученных результатов дальнейшие исследования планируются в направлении оценки вариабельности гликемии у пациентов с ожирением с использованием различных пищевых нагрузочных тестов для определения патогенетических подходов к индивидуализированной диетотерапии с учетом гетерогенности распределения жировой ткани в организме.

## ЗАКЛЮЧЕНИЕ

Оценка вариабельности гликемии у мужчин с ожирением позволяет говорить, что наличие абдоминального или подкожного типов распределения жировой ткани ассоциировано с конкретными особенностями регуляции углеводного обмена и определяет разный уровень риска развития СД2 при ПТРЖ и АТРЖ.

## ДОПОЛНИТЕЛЬНАЯ ИНФОРМАЦИЯ

Источник финансирования. Исследование проведено в рамках выполнения государственного задания учреждения.

Конфликт интересов. Авторы декларируют отсутствие явных и потенциальных конфликтов интересов, связанных с публикацией настоящей статьи.

Участие авторов. Сорокин М.Ю. — сбор материала, обработка материала; Пинхасов Б.Б. — разработка дизайна исследования, анализ полученных данных, написание текста; Лутов Ю.В. — сбор материала, обработка материала; Селятицкая В.Г. — разработка концепции исследования, критическая интерпретация результатов, написание текста, одобрение финальной версии рукописи. Все авторы внесли значимый вклад в проведение исследования и подготовку статьи, прочли и одобрили финальную версию статьи перед публикацией, выразили согласие нести ответственность за все аспекты работы, подразумевающую надлежащее изучение и решение вопросов, связанных с точностью и добросовестностью любой части работы.
